# A comprehensive point prevalence survey of the quality and quantity of antimicrobial use in Chinese general hospitals and clinical specialties

**DOI:** 10.1186/s13756-023-01334-9

**Published:** 2023-11-16

**Authors:** Yonghong Xiao, Xing Xin, Yunbo Chen, Qing Yan, The China PPS team

**Affiliations:** 1 State Key Laboratory for Diagnosis and Treatment of Infectious Diseases, National Clinical Research Center for Infectious Diseases, National Medical Center for Infectious Diseases, Collaborative Innovation Center for Diagnosis and Treatment of Infectious Diseases, The First Affiliated Hospital, Zhejiang University School of Medicine, Hangzhou, China; 2 Research Units of Infectious Disease and Microecology, Chinese Academy of Medical Sciences, Beijing, China; 3 Department of Infection Control, The Children’s Hospital, Zhejiang University School of Medicine, Hangzhou, China; 4 National Institute of Hospital Administration, National Health Commission of China, Beijing, China

**Keywords:** Antimicrobial resistance, Point prevalence survey, Chinese general hospitals

## Abstract

Antimicrobial resistance (AMR) is a serious, worldwide public health crisis. Surveillance of antimicrobial use forms part of an essential strategy to contain AMR. We aimed to conduct a national point prevalence survey (PPS) on antimicrobial use, and to compare this data with similar international surveillance programs to provide a reference for future AMR strategy development in China. Twenty general hospitals encompassing 10,881 beds and 10,209 inpatients around the country participated the survey using a standardized protocol, at 8am of someday from October 10th to November 31st, 2019. Of the patients, 37.00% (3777/10209) received antimicrobial agents, 31.30% (1630/5208) had surgical operations, and 76.63% (1249/1630) received prophylactic antibiotic. The prevalence of antimicrobial use in medical, surgical, and intensive care units (ICU) patients was 38.84% (1712/4408), 32.07% (1670/5208), and 66.61% (395/593), respectively. Of prescriptions, 5.79% (356/6151) were made in the absence of indication. The intensity of antimicrobial use was 61.25 DDDs/100 patient days, while the intensity of use in internal medicine, surgery, and ICU were 67.79, 45.81, 124.45 DDDs/100 patient days, respectively. Only 11.62% (715/6151) of prescriptions had a reason described in the patient record. Furthermore, 8.44% (210/2487), 14.19% (424/2989), and 12% (81/675) of the prescriptions in internal medicine, surgery, and ICU had a recorded indication, respectively. The review and stop date recorded for antimicrobial therapy was 43.73% (1976/4518). Of the patients, 38.07% (1438/3777) received combination therapy. The classes of antimicrobials prescribed were limited, and the proportion of prescriptions encompassed by the top 20 antimicrobial agents was 75.06% (4617/6151). The prevalence of antimicrobial use in China is close to that of Sweden, the UK, and Canada, but lower than that in India, and higher than that in Switzerland. The data described in this report indicate that the quality of antimicrobial prescriptions requires improvement in China. Further, hospitals should implement professional interventions to improve the rational use of antimicrobials.

## Introduction

Antimicrobial resistance (AMR) has become a serious worldwide public health crisis [1], and AMR control has subsequently become a global priority [2]. The World Health Organization formulated the Global Action Plan to Contain Antimicrobial Resistance in 2015 to promote international collaboration in combating AMR [3]. China is a major consumer of antimicrobial agents that also presents with high levels of AMR, and subsequently the Chinese government has prioritized control of AMR [4]. In 2016, the National Health Commission (NHC), jointly with 13 other ministries, issued the National Action Plan for AMR Containment (2016–2020), which clearly highlights the implementation of antimicrobial stewardship (AMS) to promote the rational use of antimicrobials [5]. Since 2011, the NHC has carried out a nationwide AMS campaign [6]. According to a report from the National Hospital Antibacterial Consumption Surveillance Network (NACS), the prevalence of antimicrobial use in inpatients, outpatients, and in surgical prophylaxis for clean incisions showed a downward trend following the campaign, in which the median antimicrobial use was 67.8%, 19.5%, and 97.9%, respectively, in 2010, and 40.8%, 8.5%, and 38.3%, respectively, in 2016. The national inpatient antimicrobial use intensity in medical institutions also decreased from 85.3 ± 29.8 DDDs/100 patient days in 2010 to 48.8 ± 7.7 DDDs/100 in 2013 and 48.5 ± 8.0 DDD/100 in 2016 [7, 8]. A similar phenomenon was observed in surveys of different regions and individual medical institutions [9–11].

After achieving reduction of antimicrobial use, improvements in the quality of antimicrobial agent prescription will be the focus of the AMS strategy [7, 8]. In 2018, the NHC highlighted the requirement for ‘shifting the strategy of AMS from executive administrative to multidisciplinary professional interventions. Furthermore, all healthcare institutions are required to strengthen the monitoring and surveillance of antimicrobial use, improve the process of antimicrobial prescription, and guarantee continued rational antibiotic use [12].

Point prevalence surveys (PPS) are a common epidemiological surveillance method to investigate antimicrobial use and the prevalence of resistance. In 2006, the European Surveillance of Antimicrobial Consumption Network (ESAC-Net) conducted a PPS for the use of antimicrobials in 20 countries [13]. In 2015, the University of Antwerp, Belgium, established a global-PPS for the use of antimicrobials and prevalence of bacterial resistance, which compared the results in different regions and countries around the world [14]. To facilitate comparison of data describing antimicrobial use in various regions, the WHO formulated the ‘Methodology for point prediction survey on antimicrobial use in hospitals’ in 2018, which outlined standardized surveillance protocols to study antimicrobial use over time and between hospitals, districts, countries, and regions [15]. PPS can not only investigate the consumption of antimicrobials, but also the quality of prescription practices.

In 2019, we conducted the first national pilot PPS on antimicrobial use in adult patients in general hospitals in China, which comprehensively described the quantity and quality of antimicrobial use in different hospitals, across different clinical specialties, and different infections. We subsequently compared these data with similar international surveillance studies, and provided a reference for future AMS strategies in China.

## Methods

### Enrollment of hospitals and different clinical specialties

To ensure successful implementation of the PPS, both the geographical distribution and professional situation were considered during enrollment of pilot hospitals. To accurately study the quantity and quality of antimicrobial use, clinical specialties with frequent prescription of antimicrobials were considered the main survey subjects. We expected that each medical institution would have approximately 500 patient beds to be investigated, including 250 beds in surgical departments (mandatory departments: general surgery, urology, neurosurgery, gynecology; optional or alternative departments: orthopedics, cardiothoracic surgery.), 200–250 beds of internal medicine (mandatory departments: infectious disease unit, respiratory department, hematology, and oncology; optional or alternative departments: nephrology, endocrinology, neurology, rheumatology, dermatology.), and 30–50 beds in intensive care units (ICU). No more than 100 beds were included for each department. Pediatrics and organ transplantation departments (including solid organ transplantation and stem cell transplantation) were excluded from the investigation. All inpatients in selected wards at the survey time should be investigated.

### PPS protocol

The survey was conducted at 8 am of someday from October 10th to November 31st, 2019. All questionnaires were completed within 1 week after investigation initiation. The survey protocol integrated contents recommended by the WHO and the global-PPS. Before commencing the survey, all principal investigators from participating hospitals fully discussed and agreed on implementation of the protocol. The survey protocol included patient demographics (such as gender, age, admission date, diagnosis, bacteriological results, biomarker examination, etc.), infectious diagnosis, infection type (healthcare associated infections, HAI or community acquired infections, CAI), antimicrobial use and intensity, antibiotic prescription quality including presence of indications, antibiotics class, administration route, treatment duration, compliance with guidelines, whether or not drugs were administered in combination therapy, and medication in case recording. Topical use of antimicrobial agents was not included in the PPS.

### PPS implementation

Each hospital established a PPS team based on the frequency of enrolled patients and their specialties, which was generally composed of infectious disease physicians, clinical microbiologists, clinical pharmacists, and hospital infection control specialists. The team was divided into working groups consisting of 2–3 people responsible for the study of patients in each ward. Before commencing the survey, all hospitals provided training for the investigators, including details of the study protocol, best practice for completing the questionnaire, and knowledge about antimicrobials and infections. During the study, the investigators carefully analyzed patient records and obtained clarification from on-duty medical staff on unclear contents. The PPS was approved by the Ethics Committee of the First Affiliated Hospital of Zhejiang University Medical College (approval number: 2019–093). This investigation did not involve patient personal information and was waived for requirement of informed consent.

### Statistical analysis

The double entry of data was used to establish a database, and descriptive analysis was conducted by a statistician using the SPSS software package (IBM, Chinese version 22.0). The primary analysis focused on quantity of antimicrobial use (such as prevalence, utilization intensity, surgical prophylactic use) and quality of antimicrobial use (such as presence of indication, administration route, treatment duration, combination therapy, medication recording). Antimicrobial use was defined as antimicrobial administration (i.e., receipt of at least one antibiotic) per patient. A prescription was defined as the use of one substance by one route of administration. Antimicrobial prescription rates were expressed as a percentage of patients receiving antimicrobials, or as a percentage of all antibiotic prescriptions (proportional use). The intensity of antimicrobial use was defined as the number of defined daily dosages per hundred patient days (DDDs/100 patient days). Continuous variables were expressed as the mean ± SD, or the median and range. Categorical data were expressed as proportions.

## Results

This survey included 20 general hospitals across the country, 10,881 beds (424–759 in each hospital), 10,209 inpatients (330–758 in each hospitals) including 4408 patients from eight medical specialties (108–1319), 5208 patients from nine surgical specialties (138–953), and 593 patients from the ICU. The average age of the enrolled patients was 58.15 ± 17.31 years, the proportion of males was 57.48%, and 22.58% of the patients suffered from fatal diseases (including ultimately fatal and rapidly fatal diseases defined by the McCabe classification), of which hematology, oncology, nephrology and rheumatism, tumor surgery, and ICU encompassed 70.34%, 69.93%, 50.00%, 47.83%, and 45.15% of patients, respectively. Immunosuppressed individuals accounted for 15.05% (of which 84.41% and 78.20% were in hematology and oncology departments, respectively) of patients. The proportion of males, fatal diseases, and immunosuppressed individuals were higher in internal medicine than in surgical patients. Of the patients, 3777/10209 (37.00%) received antimicrobial agents, 1630/5208 (31.30%) received surgical operations, 1249/1630 (76.63%) received prophylactic antibiotic, 1935 patients had CAI, and 387 (427 cases) were HAI (Table 1).

**Table 1:** General information and patient demographics

Patient in ward specialty		InH (n)	InP (n)	Mean age, years (SD)	Sex M/F/missing (%)	McCabe classification ①/②/③/④ (%)	Immunocom ①/②/③(%)	CAI/HAI (n)
Medicines	Infectious diseases	12	351	54.20(18.39)	63.08/35.98/0.94	80.37/11.21/2.80/5.61	83.18/12.62/4.21	162/7
	Pulmonology	20	1319	64.03(15.88)	64.26/35.45/0.31	73.91/13.31/4.16/8.63	80.56/11.02/8.42	718/10
	Hematology	17	811	52.47(19.46)	53.99/45.63/0.38	25.10/62.36/7.98/4.56	11.41/84.41/4.18	137/83
	Oncology	17	1064	60.49(13.84)	63.16/36.09/0.75	20.30/59.40/10.53/9.77	15.79/78.20/6.02	42/11
	Endocrinology	4	190	69.44(15.73)	51.86/44.44/3.70	77.78/3.70/–/18.52	77.78/3.70/18.52	7/1
	Neurology	6	363	73.80(15.90)	43.33/56.67/–	56.67/26.67/–/16.67	76.67/6.67/16.67	10/8
	Nephro-Rheumatology	5	202	54.43(16.98)	58.70/41.30/–	50.00/47.83/2.17/–	80.43/17.39/2.17	25/5
	Other internal medicines	2	108	68.43(13.09)	65.91/34.09/–	61.36/11.36/2.27/25.00	72.73/2.27/25.00	30/1
	Total of medicines	20	4408	60.88(17.43)	61.78/37.75/0.47	61.90/25.07/4.83/8.20	64.98/27.40/7.62	1131/126
Surgeries	General surgery	9	706	65.08(17.65)	58.99/39.93/1.08	70.86/14.39/1.44/13.31	76.97/11.51/11.51	71/26
	Urology	19	914	58.76(16.13)	74.66/25.15/0.19	87.43/4.64/0.19/7.74	86.65/4.06/9.28	157/7
	Neurosurgery	19	953	55.00(15.30)	61.93/38.07/–	61.33/11.78/15.71/11.18	86.40/5.74/7.85	85/105
	Gynecology	17	806	45.61(12.58)	–/100/–	97.47/0.31/0.63/1.57	93.71/3.77/2.52	9/9
	Hepatobiliary surgery	10	472	57.74(15.15)	46.06/52.76/1.18	71.65/12.20/1.97/14.17	76.77/2.76/20.47	129/15
	Orthopedics	7	393	54.22(19.20)	59.15/40.85	84.15/1.22/–/14.63	79.27/3.66/17.07	6/5
	Gastrointestinal surgery	10	553	59.24(16.03)	58.84/40.43/0.72	66.79/23.10/0.72/9.39	78.70/12.64/8.66	61/18
	Oncology surgery	2	138	57.87(14.47)	56.52/43.48/–	47.83/47.83/–/4.34	91.30/4.35/4.35	2/0
	Other surgeries	5	273	51.83(18.81)	50.00/48.75/1.25	88.75/8.75/–/2.50	96.25/2.50/1.25	26/2
	Total of surgeries	20	5208	55.36(16.54)	52.90/46.61/0.49	78.01/9.77/2.94/9.28	84.17/6.02/9.81	546/187
ICU		19	539	61.86(18.35)	64.30/35.46/0.24	44.21/15.84/29.31/10.64	83.69/12.77/3.55	258/114
Total		20	10,209	58.15(17.31)	57.48/42.06/0.46	68.43/16.35/6.23/8.99	76.60/15.05/8.35	1935/427

McCabe classification: ① non-fatal, ② ultimately fatal, ③ rapidly fatal, ④ unknown; immunocom: immunocompromised, ① no, ② yes, and ③ unknown; CAI: community acquired infection; HAI: hospital acquired infection

*ICU* Intensive care unit; *InH* Investigated hospital; *InP* Investigated patient

### Prevalence of antimicrobial use

Of the inpatients, 22.7% had an infection diagnosis (CAI: 18.95%, 1935/10209; HAI: 3.79%, 387/10209). The major infections were respiratory tract infection (RTI, 48.04%), urinary tract infection (UTI, 13.87%), and gastrointestinal/abdominal infection (9.16%). The number of patients with an infectious disease diagnosis was far lower than that of the total number of patients receiving antimicrobials (37.00%, 3777/10209). The prevalence of antimicrobial use in medical, surgical, and ICU patients was 38.84% (1712/4408), 32.07% (1670/5208), and 66.61% (395/593), respectively. There were 6151 antibacterial prescriptions in total, 2487 prescriptions for internal medicine, 2989 for surgery, and 675 for ICU. Among them, the proportion of prescriptions for treatment and prophylaxis was 67.66% (4162/6151) and 26.55% (1633/6151), respectively. Of the prescriptions, 5.79% (356/6151) lacked a recorded indication. Targeted and empiric therapy accounted for 10.43% (434/4162) and 85.25% (3548/4162) of prescriptions, respectively. Administration of medication without a recorded indication was higher in surgery patients than those in the ICU and in internal medicine. Infectious disease departments had the highest antimicrobial use without indication in internal medicine (9.14%, 31/339), and general surgery (21.41%, 79/369) and urology (12.56%, 81/645) was the highest in surgery. The proportion of patients receiving surgical clean incision was 31.29% (510/1630), and the antibiotic prophylaxis rates in surgical patients was 76.63% (1249/1630), of which ob-gynecology, orthopedics, and tumor surgery had rates higher than 90% (Tables 2 and 3).

**Table 2:** Indicators for quantity and quality of antibiotics use in internal medicine and ICU patients

Items		Internal medicine									ICU
		ID	Pulm	Hema	Onco	Endo	Neur	Ne-Rh	other	Total	
Prescriptions (n)		339	1395	419	160	30	37	55	52	2487	675
Antibiotics use rate (%)		60.4	72.78	32.31	12.31	13.68	8.26	22.77	39.81	38.79	66.27
Antibiotics use indication (%)	With	90.86	98.64	96.66	95.82	100	91.89	96.36	98.08	96.90	95.41
	Without	9.14	1.36	3.36	4.38		8.11	3.64	1.92	3.10	4.59
Therapeutics (%)	Empiric	79.06	93.26	85.68	83.12	96.67	62.16	65.45	90.38	88.30	69.13
	Targeted	9.14	3.66	10.02	11.25	3.33	37.84	29.09	3.85	7.04	22.92
	Unknown	11.80	3.08	4.30	5.62			5.45	5.77	4.66	7.94
AUI (DDDs/100 patient.day)		113.97	131.43	63.09	14.41	27.12	11.86	24.27	42.72	67.79	124.45
Guidelines (%)	Complied	83.78	88.17	93.56	78.12	73.33	91.89	89.09	88.46	87.74	81.63
	Non-complied	4.13	6.16	0.48	9.38	20.00	5.41	1.82		5.07	6.96
	Unavailable		2.01	1.19	3.12	3.33	2.70		3.85	1.69	4.59
	No guideline		0.50	0.48	2.50				1.92	0.56	
	Unknown	12.09	3.16	4.30	6.88	3.33		9.09	5.77	4.95	6.81
Therapy referring to biomarkers (%)	37.46	48.39	34.84	47.50	10.00	21.62	20.00	34.62	42.78	18.05	
Prescription reason recorded (%)		14.45	5.30	11.22	13.75	10.00	13.51	10.91	7.69	8.44	29.93
Therapeutic duration up to PPS (d)	≤ 3	27.73	32.11	29.59	38.75	26.67	24.32	25.45	51.92	31.60	31.59
	4–7	30.68	32.04	40.10	28.75	46.47	45.95	36.36	26.92	33.37	31.76
	8–14	13.57	21.08	20.53	15.00	23.33	24.32	30.91	21.15	19.86	22.75
	≥ 15	18.58	13.12	7.16	10.00		2.70			11.78	7.22
	Unknown	9.44	1.65	2.63	7.50	3.33	2.70	7.27		3.38	6.68
Administrative route (%)	Intravenous	82.01	94.06	85.20	92.64	93.33	91.89	87.27	96.08	90.69	90.71
	Oral	14.45	3.51	11.22	4.29	3.33	5.41	5.45	1.96	6.38	2.65
	Others		0.07	0.24					1.96	0.12	0.17
	Unknown	3.54	2.36	3.34	3.07	3.33	2.70	7.27		2.81	6.47
Combinatory use (%)	Single	58.96	59.06	55.13	77.86	84.62	76.67	80.43	81.82	61.74	57.97
	2 drugs	30.66	37.92	33.46	20.62	15.38	23.33	19.57	18.18	33.41	33.42
	≥ 3 drugs	10.38	3.03	11.41	1.52					4.85	8.61
Review or stop recorded (%)		51.62	26.74	43.44	46.88	46.67	32.43	56.36	9.62	34.86	44.98

*ID* Infectious disease; *Pulm* Pulmonary department; *Hema* Hematology department; *Onco* Oncology department; *Endo* Endocrinology department; *Neur* Neurology department; *Ne-Rh* Nephrology & Rheumatology department; *ICU* Intensive care unit; *RTI* Respiratory tract infections; *NA* Non-applicable; *FN* Fever with neutropenic; *FUO* Fever unknown origin; *MP* Medicine prophylaxis; *GI/IA*, Gastroenterological infection and intra-abdominal infection; *SST/BJ* Skin and skin structure, and bone and joint infections; *UTI* Urinary tract infection; *AUI* Antibiotic use intensity

**Table 3:** Indicators for quantity and quality of antibiotic use in surgery patients

Items		GenS	Urol	NeuroS	Gyn	HepaB	Ortho	GiS	OncoS	Others	Total
Prescriptions (n)		369	645	431	511	327	185	418	26	77	2989
Antibiotics use rate (%)		31.59	50.98	21.62	23.95	45.34	22.14	36.17	15.94	18.68	31.91
Surgical type (%)	Clean	9.52	23.37	64.29	22.92	16.09	69.48	14.22		40.91	31.29
	Contaminated	29.25	37.38	8.65	68.06	48.85	10.39	62.84	77.78	25.00	39.57
	Infected	7.48	0.62	1.13	1.74	8.62	9.74	1.83		4.55	3.50
	Unknown	53.74	38.63	25.94	7.29	26.44	10.39	21.10	22.22	29.55	25.64
Antibiotics use purpose (%)	Therapeutics	52.57	55.19	43.62	25.05	43.73	11.35	35.41	26.92	57.14	41.12
	Prophylaxis	26.02	32.25	51.04	74.17	51.07	81.62	60.77	73.08	23.38	50.59
	Unknown	21.41	12.56	5.34	0.78	5.20	7.03	3.83		19.48	8.30
Therapeutics (%)	Empiric	85.35	87.19	61.14	89.39	93.75	50.00	87.81	85.70	89.83	83.34
	Targeted	2.57	7.78	33.65	6.08	3.13	38.25	6.092	14.30	3.39	10.22
	Unknown	12.08	5.03	5.21	4.53	3.13	11.75	6.09		6.77	6.44
Surgical prophylaxis (%)		57.14	59.50	76.69	95.14	76.44	95.45	83.49	94.44	38.64	76.63
AUI (DDDs/100 patient.day)		43.82	73.67	31.42	43.23	67.68	27.70	62.79	40.19	23.74	45.81
Guidelines(%)	Complied	73.71	78.14	83.29	82.78	81.96	90.81	77.03	100	76.62	80.33
	Non-complied	9.49	15.50	10.90	11.15	11.62	4.32	12.44		3.90	11.38
	Unavailable	6.50	1.86	0.93		0.92		6.22		14.29	2.68
	No guideline	1.36	0.62	0.93		0.92	1.08			1.30	0.64
	Unknown	8.94	3.88	3.94	6.07	4.59	3.78	4.31		3.90	4.98
Therapy referring to biomarkers (%)		28.46	40.00	20.42	14.48	21.10	2.16	19.38	23.08	46.75	24.12
Prescription reason recorded (%)		29.81	17.36	6.73	7.83	14.98	2.16	10.29	23.08	40.26	14.19
Therapeutic duration up to PPS (d)	≤ 3	32.96	33.15	23.69	52.26	38.12	29.43	29.88	71.43	38.98	36.89
	4–7	33.33	42.95	21.32	34.84	24.99	32.37	35.34	28.57	38.98	32.23
	8–14	13.19	17.99	22.75	10.61	27.51	11.75	21.95		13.56	17.40
	≥ 15	18.31	2.68	27.96	1.51	6.25	17.63	9.15		5.09	10.50
	Unknown	2.20	3.22	4.27	0.77	3.13	8.81	3.67		3.39	2.97
Administrative route (%)	Intravenous	96.00	94.20	93.54	83.71	97.48	91.27	97.14	97.37	95.38	93.66
	Oral	1.23	3.48	1.90	16.01	0.31	5.56	1.04		1.54	4.04
	Others		0.34				0.79		2.63		0.16
	Unknown	2.77	1.99	4.56	0.28	2.20	2.38	1.82		3.08	2.14
Combinatory use (%)	Single	63.84	74.95	76.56	40.21	64.65	58.62	41.79	36.36	78.43	63.05
	2 drugs	29.91	21.63	21.53	46.91	27.91	39.08	40.30	59.09	17.65	30.00
	≥ 3 drugs	6.25	3.43	1.92	12.88	7.45	2.30	17.92	4.55	3.92	6.94
Stop or review recorded (%)		58.27	39.84	19.72	16.83	25.08	5.95	16.99	23.08	61.04	44.94

*GenS* General surgery; *Urol* Urology; *NeurS* Neurosurgery; *Gyn* Gynecology; *HepaB* Hepatobiliary surgery; *Orth* Orthopedics; *GiS* Gastrointestinal surgery; *OncoS* Oncological surgery; *RTI* Respiratory tract infection; *NA* Non-applicable; *FUO* Fever unknown origin; *GI/IAI* Gastroenterological infection and intra-abdominal infection; *SST&BJ* Skin and skin structure, and bone and joint infections; *UTI* urinary tract infection; *GUM* Male genital infection; *CNS* Central nerve system infection; *GY* Ynecological infection; *AUI* Antibiotic use intensity

The overall intensity of antimicrobial use in hospitals was 61.25 DDDs/100 patient days, while that of internal medicine, surgery, and the ICU was 67.79, 45.81, and 124.45 DDDs/100 patient days, respectively. The intensity of use in infectious disease departments, respiratory departments, urology, hepatobiliary pancreatic surgery, and gastrointestinal surgery was higher than that of internal medicine or surgery as a whole.

### Quality of antimicrobial prescriptions

The overall quality of antimicrobial prescriptions was low. Only 11.62% (715/6151) of prescriptions had a reason described in the patient record. Of prescriptions, 8.44% (210/2487), 14.19% (424/2989), and 12% (81/675) had a described reason in internal medicine, surgery, and the ICU, respectively. Worryingly, in respiratory departments, orthopedic, neurosurgery, and ob-gynecology rates of described prescription indication were less than 10%. Considering cases of therapeutic antibiotic use, empirical, targeted, and purpose unclarified therapeutical administration accounted for 84.33% (3810/4518), 10.03% (453/4518), and 5.64% (255/4518), respectively. Rates of empiric antimicrobial therapy in internal medicine, surgery, and the ICU were 89.21% (2150/2487), 83.34% (1231/1477), and 69.13% (383/554), respectively. Furthermore, over 90% of antimicrobial therapies were empiric in respiratory departments, endocrinology, hepatobiliary, and pancreatic surgery. The review and stop dates recorded for antimicrobial therapy was 43.73% (1976/4518), and that of internal medicine, surgery, and ICU was 34.86% (867/2487), 58.23% (860/1477), and 44.94% (249/554), respectively. Further, the respiratory department (26.74%) and orthopedics (5.95%) were the lowest specialties in internal medicine and surgery. During the survey, 65.41% (2988/4518) of patients were treated with antimicrobials for less than 7 days, with 64.97% (1616/2487), 69.12% (1021/1477), and 63.35% (351/554) of patients in medical, surgical, and ICU departments, respectively, receiving less than 7 days of therapy. Of the therapeutic drugs, 91.98% (5128/5575) was administered intravenously. The frequency of combination therapy was 38.07% (1438/3777), with internal medicine, surgery, and ICU presenting with combination therapy rates of 38.26% (655/1712), 36.95% (617/1670), and 42.03% (166/395), respectively. Only 41.72% (1885/4518) of patients were treated with antibiotics following reference to microbiological or biomarker examination (Tables 2 and 3).

### Antimicrobial class prescribed

**Fig. 1:**
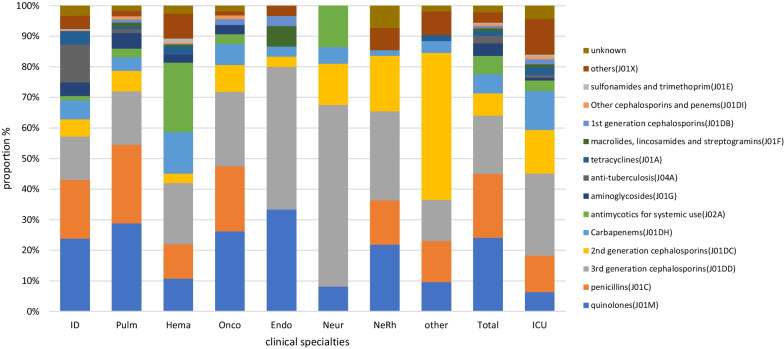
Proportion of prescribed antibiotic classes for systemic use in internal medicine and intensive care units. ID, Infectious disease; Pulm, Pulmonary department; Hema, hematology department; Onco, oncology department; Endo, endocrinology department; Neur, Neurology department; Ne-Rh, Nephrology & Rheumatology department; ICU, intensive care unit

**Fig. 2:**
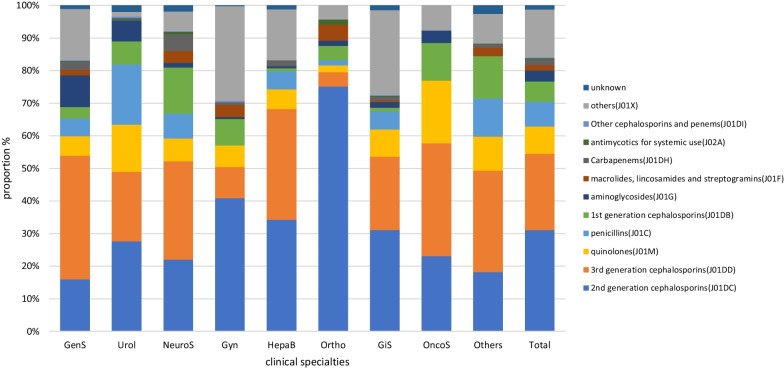
Proportion of prescribed antibiotic classes for systemic use in surgeries. GenS, general surgery; Urol, urology; NeurS, neurosurgery; Gyn, gynecology; HepaB, hepatobiliary surgery; Ortho, orthopedics; GiS, gastrointestinal surgery; OncoS, oncological surgery

The classes of specific antimicrobial agents prescribed was limited. The majority of antimicrobial classes prescribed (69.00% of total prescriptions) encompassed third generation cephalosporins (3GC) and their combination with β-lactamase inhibitors (CLI) (22.05%, 1356/6151), second-generation cephalosporins (2GC; 19.61%, 1206/6151), quinolones (14.50%, 892/6151), and broad-spectrum penicillins combined with β-lactamase inhibitors (BPLI; 13.41%, 825/6151). The antimicrobial classes most frequently used in internal medicine, surgery, and the ICU were quinolones (24.13%, 600/2487), 2GC (31.33%, 930/2989), and 3GC (26.96%, 182/675), respectively. 2GC was used most frequently (43.78%, 715/1633) for surgical prophylaxis, but 3GC (18.25%, 298/1633) and nitroimidazoles (12.25%, 200/1633) also accounted for a large proportion of prescriptions. Carbapenems were mainly used in the ICU, hematology, respiratory departments, infectious disease departments, and neurosurgery. Antifungal agents for systemic use were mainly used in hematology and ICU (Figs. 1 and 2).

**Fig. 3:**
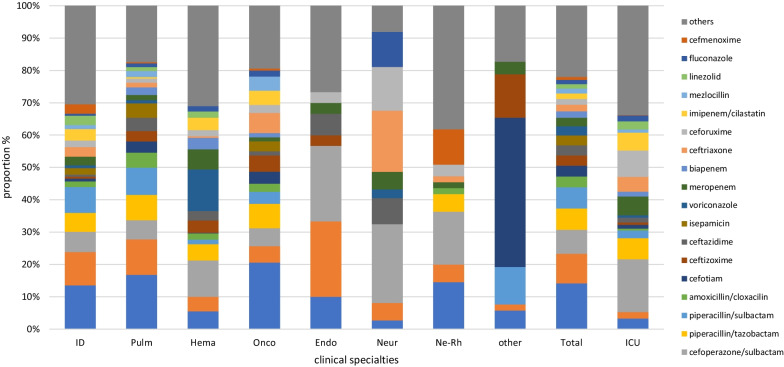
Proportion of the most frequently prescribed 20 antibiotic agents for systemic use in internal medicines and intensive care units. ID, Infectious disease; Pulm, Pulmonary department; Hema, Hematology department; Onco, Oncology department; Endo, Endocrinology department; Neur, Neurology department; Ne-Rh, Nephrology & Rheumatology department; ICU, Intensive care unit

**Fig. 4:**
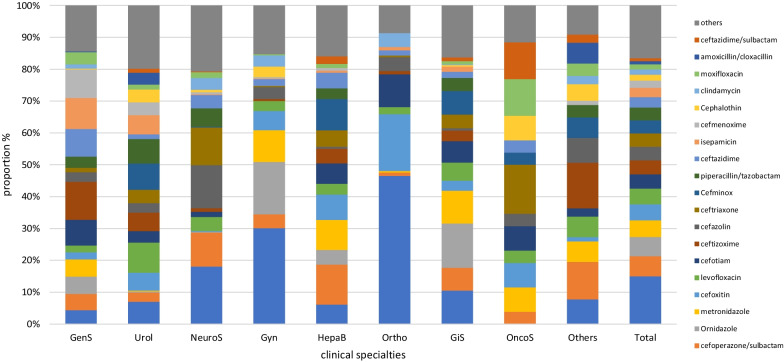
Proportion of the most frequently prescribed 20 antibiotic agents for systemic use in surgeries. GenS, General surgery; Urol, Urology; NeurS, Neurosurgery; Gyn, Gynecology; HepaB, Hepatobiliary surgery; Ortho, Orthopedics; GiS, gastrointestinal surgery; OncoS, oncological surgery

The proportion of the top 20 antimicrobial agents prescribed was 75.06% (4617/6151), encompassing 77.97% (1932/2478), 83.64% (2500/2989), and 66.21% (435/657) of patients in internal medicine, surgery, and the ICU, respectively. The frequency of prescriptions of the top four individual antimicrobial agents were cefuroxime (8.93%), levofloxacin (8.50%), cefoperazone/sulbactam (7.87%), and piperacillin/tazobactam (5.33%), which accounted for 30.63% in total. The top four agents accounted for 37.27% of prescriptions in internal medicine (levofloxacin, 14.15%; moxifloxacin, 9.13%; cefoperazone/sulbactam, 7.44%; and piperacillin/sulbactam, 6.55%), 32.55% in surgery (cefuroxime, 15.02%; cefoperazone/sulbactam, 6.32%; ornidazole, 6.02%; and metronidazole, 5.19%), and 36.75% in the ICU (cefoperazone/sulbactam, 16.30%; cefuroxime, 8.15%; piperacillin/sulbactam, 6.52%; and meropenem, 5.78%). The composition of antimicrobial use across clinical specialties varied markedly (Figs. 3 and 4).

**Fig. 5:**
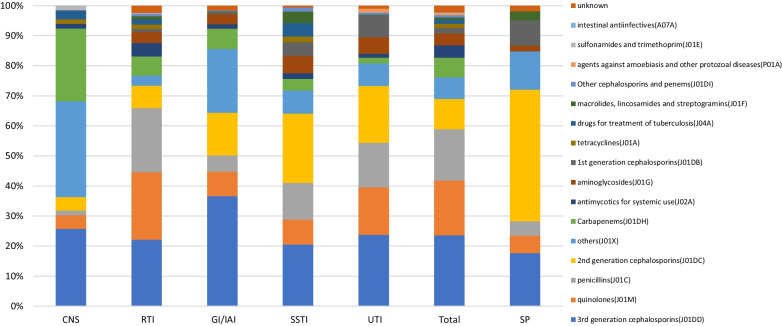
Proportion of prescribed antibiotic classes for systemic use in different infections and for surgical prophylaxis. CNS, Central nerve system infection; RTI, Respiratory tract infection; GI/IAI, Gastroenterological infection and intra-abdominal infection; SSTI Skin and skin structure infections; UTI Urinary tract infection; SP Surgical prophylaxis

**Fig. 6:**
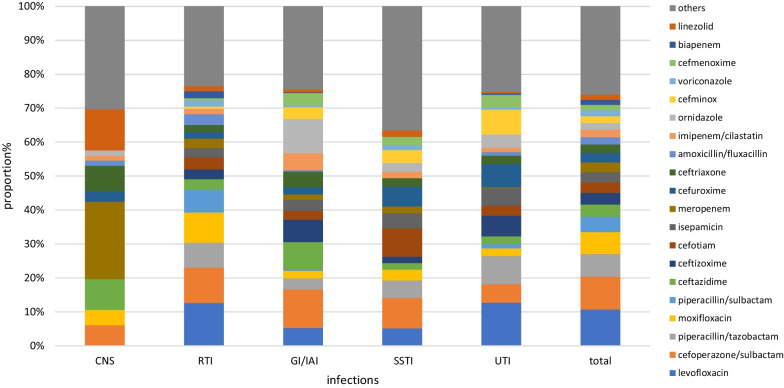
Proportion of the most frequently prescribed 20 antibiotic agents for systemic use in different infections. CNS, Central nerve system infections; RTI, Respiratory tract infections; GI/IAI, Gastroenterological infection and intra-abdominal infection; SSTI, Skin and skin structure infections; UT, Urinary tract infection

**Fig. 7:**
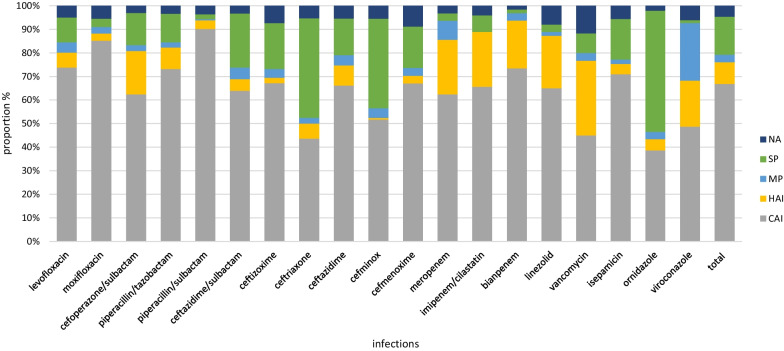
Proportions of indications for the most frequently prescribed restricted or special antimicrobials. CAI, Community acquired infection; HAI, Healthcare associated infection; MP, Prophylaxis in internal medicine; SP, Surgery prophylaxis; NA, Not applicable

The top three antibacterial drugs used to treat CAI were 3GC [24.27%, including cefoperazone/sulbactam (8.90%)], quinolones [19.54%, including levofloxacin (11.38%)], BPLI [19.33%, including piperacillin/tazobactam (7.07%)]. By contrast, the main drugs used for treatment of HAI were 3GC [28.48%, including cefoperazone/sulbactam (18.70%)], carbapenems [13.71%, including meropenem (6.09%)] and BPLI [9.92%, including piperacillin/tazobactam (6.30%)]. With the exception of central nervous system infections (CNSI), the prescriptions of antibacterial drugs for other infections followed a comparable pattern. The main classes of antibacterial agents used for treatment of CNSI were carbapenems (24.24%, 16/66) and 3GC (25.76%, 17/66) (Figs. 5 and 6). Among the 20 most commonly prescribed antibacterial agents, 16 were highlighted as restricted by the AMS strategy. Prescriptions with non-therapeutic purposes accounted for 22.50% (784/3484), including prescription of ceftriaxone, cefminox, ceftazidime/sulbactam, ornidazole, voriconazole, and vancomycin in 49.03% (101/206), 44.81% (69/145), 29.69% (19/64), 56.61% (107/189), 31.71% (26/82), and 23.33% (14/60) of cases, respectively (Fig. 7).

## Discussion

China is one of the main consumers of antimicrobial agents, with relatively high levels of associated AMR [1, 16]. Despite this, antimicrobial use in hospitals has decreased remarkably after implementation of a national AMS campaign launched by the Ministry of Health in 2011 [6, 7]. According to a report from the NACS in 2017, the prevalence of antimicrobial use in inpatients in general hospitals was 36.9%, use in surgical prophylaxis was 66.2%, and the intensity of antimicrobial use was 45.7 DDDs/100 patient days [7]. The cost of antimicrobial procurement in medical institutions decreased from 22.3% of total medicine expenditure in 2010 to 12.1% in 2016 [8]. This trend was observed across different regions and hospitals in China. For example, the prevalence and intensity of antimicrobial use was 34.65% and 37.38 DDDs/100 patient days, respectively, in a tertiary hospital in Beijing in 2016 [9], while they were 58.05% and 58.26 DDDs/100 patient days in Guangzhou in 2017 [17]. The consumption of antimicrobials in specialized hospitals also decreased prominently [11]. In accordance with the long-term ‘two steps and two hands’ AMS strategy proposed by the NHC in 2018, the major AMS strategy should transition from executive administrative to professional interventions, with the main focus on improving the quality of antimicrobial use by constantly enhancing professional capability [4, 12].

The NACS was established in China in 2005 and adopted a sampling method for AMR surveillance. Each member hospital randomly samples 15 medical records of discharged non-operating patients and 15 surgical patients every month, reflecting 360 patients throughout the year. The main survey outcomes are the prevalence and intensity of antimicrobial use and surgical prophylaxis. This survey method is different from that recommended by the WHO and the internationally recognized PPS protocol. Therefore, these results could not be directly compared [7, 14, 15]. To standardize the monitoring of antimicrobial use internationally, we implemented the first national PPS in Chinese general hospitals. In this survey, 20 general hospitals in different regions across the country were enrolled, and the WHO and global-PPS methods were integrated into the protocol. The PPS was successfully implemented in our country, which lays a foundation for expanding enrollment of survey hospitals and sets a precedent for eventually conducting the survey nationwide.

We found that after nearly 10 years of a national AMS campaign, the use of antimicrobials in general hospitals has significantly decreased. The prevalence, intensity of antimicrobial use, and frequency of surgical prophylaxis were 37.00%, 61.25 DDDs/100 patient days, and 74.97%, respectively, which was far lower than the 67.30%, 85.90 DDDs/100 patient days, and 95.40%, respectively, reported by the NACS in 2010 [6, 7]. These data were similar to those reported by the European PPS in 2011 (37.4%); slightly higher than those in Europe in 2016 (32.9%); far lower than Singapore and some European countries such as Italy, Spain, Portugal, and Greece; similar to Norway, Sweden, and the United Kingdom (UK); slightly higher than Germany, France, and Canada [13, 18–20]; and lower than that in the United States (US) in 2015 (49.6%) [21]. The intensity of antimicrobial use was higher than that reported in a European PPS in 2016 (46 DDDs/100 patient days), lower than that of Italy and the UK, higher than Western and Northern European countries, and similar to Taiwan in 2015 [18]. Compared with a global-PPS in 2015, the prevalence of antimicrobial use in Chinese medical institutions is similar to that in the Americas, Southeast Asia, and Southern Europe, but lower than that in Central and Western Asia, Africa, and South America [14]. The frequency of surgical prophylaxis (76.63%) was much higher than the global-PPS results published in 2015 (17.8%), and higher than that in Europe in 2016 (54.2%) [14, 18]. Antimicrobial consumption in Chinese hospitals is generally close to that in Sweden and the UK, and higher than in Switzerland [22].

While the use of antibiotics is decreasing, improving prescription quality is also an important feature of rational antibiotic use. Our survey found that the quality of antimicrobial prescriptions in medical institutions and across clinical specialties required significant improvement. Only 11.62% of antimicrobial use described reasons for prescription in the patient record, which was far lower than that in Europe (80.2%) and Canada (87.3%) in 2016. The frequency of combination therapy was 38.07%, higher than that described in a European PPS in 2016 (29.04%), Singapore (25.9%) and Switzerland (22.3%) [18–20, 23]. Targeted therapy (10.03%) was lower than that of the global-PPS (76.9%) and Canada (39.4%). Prescriptions were predominantly for intravenous antibiotics (92.02%), which was much higher than that of Europe in 2012 (79.4%), the global-PPS (71.4%), and Singapore (59%), but similar to Romania and Greece [14, 18, 19]. Only compliance to the guidelines (83.47%) was higher than that of the global-PPS (77.4%), and those of community-acquired pneumonia (CAP), and urinary tract infection (UTI) prescriptions in US and Latin America [14, 21, 24, 25].

The survey revealed that medical institutions preferred to use restricted and special classes of antimicrobial agents. Among the 20 most prescribed antimicrobial agents, 16 belonged to restricted and special antibacterial drugs defined by the AMS strategy [23]. Of the drugs, 22.50% were for non-therapeutic purposes, encompassing rates of use of ceftriaxone, cefminox, ornidazole, voriconazole, and vancomycin of 49.03%, 44.81%, 56.61%, 31.71%, and 23.33%, respectively. Although cefuroxime was the most common antibiotic prescribed for surgical prophylaxis, rates of prescription of 3GC, nitroimidazoles, and quinolones were 17.70%, 12.25%, and 5.81%, respectively. The top three antimicrobial agents used to treat CAI were cefoperazone/sulbactam (8.90%), levofloxacin (11.38%), and piperacillin/tazobactam (7.07%), a similar pattern to that observed in Eastern Europe and Latin America [7, 14, 22].

The classes of antimicrobials prescribed in general hospitals were also limited, with low levels of pharmacological diversity. 3GC, CLI, 2GC, quinolones, and BPLI accounted for nearly 70% of the total prescriptions. The top 20 antibacterial agents prescribed accounted for 75.06% of the total, comparable with that described in Eastern European countries by the global-PPS, as well as South Korea and Taiwan [14, 22, 26]. Penicillins remain a cost-effective choice for treatment of infections caused by susceptible bacteria. With the exception of BPLIs such as piperacillin/tazobactam, the use of first generation penicillins was rare, far lower than that in Northern Europe, Western Europe, Southeast Asia, and the Americas [14, 18]. This may reflect the requirement in China for a penicillin skin test before use of all penicillins, and the relatively high rates of bacterial resistance. Use of the penicillin skin test should be determined by improving drug quality and data in large populations, confirming the real incidence of penicillin anaphylaxis [7, 27, 28].

The most commonly used antimicrobials were quinolones in internal medicine (24.66%), 2GC (31.50%) in surgery (mainly for prophylaxis), and 3GC in the ICU (28.17%). There is room for improvement in the use of antimicrobials across various clinical specialties, improving proportions of empirical use, frequency of combination therapy, record of drug use, and preference for intravenous administration. Worryingly, infectious disease and respiratory departments, which should be the leading specialties promoting AMS practices in medical institutions [5], were comparable to other specialties when considering quality of antibiotic prescription. For example, the prevalence of antimicrobial use in respiratory departments was 72.78%, which was higher than that of the ICU. Further, respiratory departments were the departments within internal medicine with the lowest rate of descriptions with recording reasons for antibiotic use (5.30%). The intensity of use of antimicrobials in respiratory and infectious diseases departments was 131.43 and 113.97 DDDs/100 patient days, respectively. The proportion of prescriptions that referred to microbiological examinations and biomarkers in the infectious diseases department (37.46%) was lower than that in the overall internal medicine cohort (42.78%), and the proportion of combination therapy in the respiratory and infectious diseases departments was higher than that in the overall internal medicine cohort. Worryingly, these rates were much higher than those reported by the global-PPS, Canada, and Latin America [13, 20, 25]. These data indicate that medical institutions may struggle to promote good AMS practices. The main reason may be due to the disease composition and scope of professional work in the two departments. In the past, infectious disease physicians in China have been mainly engaged with the diagnosis and treatment of legally reportable communicable diseases (such as viral hepatitis, tuberculosis, AIDS, etc.), and they did not have sufficient practice in the management of bacterial infections, or experience in AMS. This is in stark contrast to their colleagues in the US, Europe, and other countries [29]. Furthermore, Chinese respiratory physicians mainly diagnose and treat lung tumors (the predominant carcinoma and top disease observed in these department in our country), which may impede AMS practices [30]. To this end, the NHC has called for all medical institutions to improve their infectious diseases departments and promote the leading role of infectious physicians in implementing AMS practices [5, 25].

This is the first time that we have successfully implemented a PPS, and defined the quantity and quality of antimicrobial use in general hospitals and across clinical specialties in China. This PPS mainly enrolled general hospitals with better AMS practice, and investigated the clinical specialties with frequent use of antimicrobials. The survey did not investigate all patients in the hospital, as this may overestimate the use of antimicrobials. Although all investigators were trained on implementation of the protocol and investigation procedures, some data collection deficiencies are unavoidable in individual medical institutions, which may have also impacted the results.

## Conclusions

After more than 10 years implementing a special national AMS campaign, the consumption of antimicrobials in medical institutions in China has decreased. The prevalence of antimicrobial use is now close to that of Sweden, the UK, and Canada; lower than that in India; and higher than that in Switzerland [14, 23]. However, close attention to the quality of antimicrobial prescriptions is required. AMS practices should be improved in medical institutions, including establishment of professional teams and training initiatives for infectious disease physicians, allowing them to take responsibility of AMS in their institute. Hospitals should regularly carry out PPS for antimicrobial use and implement professional intervention to improve understanding of infectious diseases and antimicrobials, as well as rational antibiotic use.

## Data Availability

The datasets for the study are available from the corresponding author upon request.

## Abbreviations

AMR: Antimicrobial resistance
PPS: Point prevalence survey
NHC: National Health Commission
AMS: Antimicrobial stewardship
NACS: National hospital antibacterial consumption surveillance network
ESAC-Net: European surveillance of antimicrobial consumption network
HAI: Healthcare associated infections
CAI: Community acquired infections
RTI: Respiratory tract infection
UTI: Urinary tract infection
BPLI: Broad-spectrum penicillins combined with β-lactamase inhibitors
3GC: Third generation cephalosporins
2GC: Second generation cephalosporins
ICU: Intensive care units
